# Knowledge and practice of Brazilian pediatricians on gastroesophageal reflux disease in infants

**DOI:** 10.1016/j.rpped.2014.11.005

**Published:** 2015-03

**Authors:** Ana Cristina Fontenele Soares, Carla Lima de Freitas, Mauro Batista de Morais

**Affiliations:** Universidade Federal de São Paulo, São Paulo, SP, Brazil

**Keywords:** Gastroesophageal reflux, Vomiting, Infant, Infant formula, Professional practice, Health knowledge, attitudes, practice

## Abstract

**OBJECTIVE:**

To assess the knowledge and practice of pediatricians about infants with physiological reflux and gastroesophageal reflux disease.

**METHODS:**

140 pediatricians were interviewed during two scientific events in 2009 and 2010. The questions referred to two clinical cases of infants. One with symptoms of infant regurgitation (physiological reflux) and another with gastroesophageal reflux disease.

**RESULTS:**

Among 140 pediatricians, 11.4% (n=16) and 62.1% (n=87) would require investigation tests, respectively for infant regurgitation (physiological reflux) and gastroesophageal reflux disease. A series of upper gastrointestinal exams would be the first requested with a higher frequency. Medication would be prescribed by 18.6% (n=6) in the case of physiological reflux and 87.1% (n=122) in the case of gastroesophageal reflux disease. Prokinetic drugs would be prescribed more frequently than gastric acid secretion inhibitors. Sleeping position would be recommended by 94.2% (n=132) and 92.9% (n=130) of the respondents, respectively for the case of physiological reflux and gastroesophageal reflux disease; however, about half of the respondents would recommend the prone position. Only 10 (7.1%) of the pediatricians would exclude the cow's milk protein from the infants' diet.

**CONCLUSIONS:**

Approaches different from the international guidelines are often considered appropriate, especially when recommending a different position other than the supine and prescription of medication. In turn, the interviews enable us to infer the right capacity of the pediatricians to distinguish physiologic reflux and gastroesophageal reflux disease correctly.

## Introduction

Vomiting and regurgitation frequently occur in infants, particularly in the first semester of life.^1^ Most cases are caused by physiological reflux.^2^^,^ 3 On the other hand, gastroesophageal reflux disease (GERD) is characterized by varied and nonspecific clinical manifestations, not necessarily restricted to the digestive tract.^3^ The borderline between infant regurgitation and gastroesophageal reflux disease is not always easily defined and its differentiation is often a challenge when assisting the infant.^2,3^ In this context, there is a growing concern about excessive test requests and medication prescriptions for healthy infants who have regurgitation that is not caused by gastroesophageal reflux disease.^2^^-^ 5 On the other hand, GERD has varied symptoms, and if not properly managed, it can cause morbidity.^2^^-^ 4

Guidelines for the care of infants with gastroesophageal reflux have been published in recent decades, with changes in the diagnostic and therapeutic recommendations.^2^^,^ 3 ^,^ 6 ^,^ 7 In 2007, an article was published based on surveys carried out with professionals in North America, showing that despite the existence of several guidelines, many infants with physiological gastroesophageal reflux are still being treated in North America as if they had GERD.^8^

Considering that surveys with professionals allow the guidance of continuing medical education programs, this study aimed to evaluate the knowledge and practice of Brazilian pediatricians when treating infants with physiological reflux (infant regurgitation) and gastroesophageal reflux disease. 

## Method

Data collection was carried out in two scientific events held in October 2009 and March 2010. A total of 140 physicians were interviewed, 121 (86.4%) of which were females, after being randomly invited to participate. Regarding the year of graduation from Medical School, 56 had graduated after 2005, 34 had graduated between 2000 and 2005, and 50 had graduated before 2000. All the participants signed the Free and Informed Consent Form after being informed about the purpose of the research. The project was approved by the Ethics Committee of Universidade Federal de São Paulo - Hospital São Paulo. 

Regarding the place where the physicians worked, 107 (76%) respondents worked in the Southeast region, mostly in the state of São Paulo (66 in the capital city and 32 in the countryside). The other participants worked in the Northeast (n=8; 6%), South (n=9; 6.5%), North (n=7; 5.5%) and Central-West (n=8; 6%) regions.

The questions were formulated based on clinical scenarios similar to those previously used in the literature:^8^

Clinical scenario 1 (expectation that a diagnosis of regurgitation in infants or "physiological reflux" was established): "involuntary vomiting 2-4 times a day in a 2 month-old infant receiving infant formula, at the 90^th^ percentile for weight and height. No other clinical manifestations."

Clinical scenario 2 (expectation that a diagnosis of gastroesophageal reflux disease was established): "five-month-old infant receiving infant formula. The infant has frequent regurgitation since birth and for 2 months has been showing signs of irritability and difficulty gaining weight. She showed partial improvement with postural measures." 

The following questions were formulated for each of the two clinical scenarios, and respondents were asked to answer them according to their medical practice: 1- Would you request any diagnostic tests? If the answer is yes, what is the first test?; 2 - Would you prescribe any medication? If yes, what would be your first prescription?; 3 - Would you modify the diet? If yes, what is your first recommendation?, and 4 - What sleeping position in the crib would you recommend? Alternatives were offered for all questions as shown in Tables 1 and 2 of the Results section. The interview files included brand names of dietetic products and medications.

**Table 1:** Practice of pediatricians for a clinical scenario compatible with regurgitation in infants (physiological reflux) and another clinical scenario compatible with gastroesophageal reflux disease in the first semester of life regarding the indication for diagnostic tests and medication prescription.

	Clinical Scenario 1: ‘physiological reflux’ (n=140)	Clinical Scenario 2: GERD (n=140)
Do you request diagnostic tests? What is the 1st test?		
No	124 (88.6%)	53 (37.9%)
Yes, what is the 1st test?	16 (11.4%)	87 (62.1%)
Contrast x-ray of esophagus, stomach and duodenum	7 (43.8%)	48 (55.1%)
24-hour esophageal pH-metry	5 (31.3%)	30 (34.5%)
Radionuclide study (scintigraphy)	1 (6.2%)	4 (4.6%)
Ultrasound for GER assessment	2 (12.5%)	2 (2.3%)
Upper digestive endoscopy with biopsy	—	2 (2.3%)
Esophageal manometry	—	(1.2%)
Plain abdominal x-ray	1 (6.2%)	—
Do you prescribe any medication?		
No	114 (81.4%)	18 (12.9%)
Yes, what is the first prescription?	26 (18.6%)	122 (87.1%)
Metoclopramide	1 (3.8%)	—
Bromopride	13 (50.0%)	28 (23.0%)
Domperidone	12 (46.2%)	43 (35.2%)
Bromopride + ranitidine or omeprazole	—	2 (1.6%)
Domperidone + ranitidine	—	24 (19.7%)
Domperidone + omeprazole	—	5 (4.1%)
Ranitidine	—	15 (12.3%)
Omeprazole	—	4 (3.3%)
Lansoprazole	—	1 (0.8%)

GERD, gastroesophageal reflux disease; GER, gastroesophageal reflux.

**Table 2:** Practice of pediatricians compatible with a clinical scenario of regurgitation in infants ("physiological reflux") and another clinical scenario compatible with gastroesophageal reflux disease in the first semester of life regarding the recommendation for position in the crib and change in formula.

	Scenario 1: “Physiological reflux” (n=140)	Scenario 2: GERD (n=140)
Would you recommend change in position?		
No	8 (5.8%)	10 (7.1%)
Yes, what is the position?	132 (94.2%)	130 (92.9%)
Prone position	1 (0.7%)	3 (2.4%)
Prone position with 30° elevation	29 (22.0%)	32 (24.6%)
Supine position	4 (3.0%)	5 (3.8%)
Supine position with 30° elevation	61 (46.2%)	54 (41.5%)
Right lateral decubitus	5 (3.8%)	3 (2.4%)
Right lateral decubitus with 30° elevation	7 (5.3%)	7 (5.4%)
Left lateral decubitus	8 (6.1%)	8 (6.1%)
Left lateral decubitus with 30° elevation	17 (12.9%)	18 (13.8%)
Any position	—	—
Would you recommend a change in formula?		
No	98 (70.0%)	35 (25.0%)
Yes, what change?	42 (30.0%)	105 (75.0%)
Add a thickening agent to the bottle	8 (19.0%)	25 (23.8%)
Anti-regurgitation infant formula	33 (78.6%)	70 (66.7%)
Formula with partially hydrolyzed protein	—	4 (0.3%)
Formula with extensively hydrolyzed proteins	—	—
Formula with amino acids	—	—
Formula with soy protein	2 (0.4%)	6 (0.6%)

GERD, gastroesophageal reflux disease.

Considering the guideline of NASPGHAN/ESPGHAN^3^ (North American Society for Pediatric Gastroenterology, Hepatology and Nutrition/European Society for Pediatric Gastroenterology, Hepatology and Nutrition), published in 2009, which emphasizes the association between cow's milk allergy and gastroesophageal reflux in children, the following questions were formulated: "In infants, can gastroesophageal reflux disease be secondary to cow's milk protein allergy?" If the answer is yes: 1 - Do you request any diagnostic test? If yes, what is the first test to be requested?; 2 - For an infant younger than 6 months of age, receiving infant formula with suspected gastroesophageal reflux disease secondary to cow's milk protein allergy, which of the following dietary options do you initially prescribe? 

Statistical analysis was carried out using Epi-Info software, release 3.2.2 (Atlanta, GA, USA) to calculate the chi-square test.

## Results

Table 1 shows the results for the questions related to clinical scenarios 1 and 2 regarding diagnostic tests required and medication prescription. For an infant with symptoms compatible with "physiological reflux", 88.6% of respondents would request no tests, and 18.6% would prescribe one prokinetic medication. For an infant with symptoms suggestive of GERD (clinical scenario 2), the proportions of respondents who would request tests and prescribe medications are higher. The initially requested test, in most cases, is the contrast radiography of the esophagus, stomach and duodenum. In the presence of a scenario compatible with GERD, 87.1% of respondents prescribe drugs, with a predominance of prokinetics and ranitidine.

Regarding the recommended position for the child in the crib (Table 2), we observe that approximately 95% of respondents advocate change for both clinical scenarios. However, positions that are different than the currently recommended (supine) would be suggested by a significant number of respondents (54.4% for infants with "physiological reflux" and 42.1% for infants with symptoms compatible with GERD).

Industrialized thickened formula or adding thickener to formula is the predominant recommendation, emphasizing that the change in the type of bottle is recommended more frequently in the clinical scenario corresponding to GERD (Table 2). On the other hand, it should be noted that only ten respondents would exclude cow's milk protein from the diet as the first dietary change for the infant with symptoms compatible with gastroesophageal reflux disease (Table 2).

Regarding the question about the possibility of gastroesophageal reflux disease being secondary to cow's milk allergy, 105 (75.0%) of respondents answered yes, 16 (11.4%) answered no, and 19 (13.6%) said they are similar clinical manifestations, but are caused by different diseases. 

Of the 140 respondents, 86 (82.0%) would request some type of test, with the following being the initially requested ones: measurement of specific IgE against cow's milk would be requested by 44 (51.2%), serum total IgE by 24 (27.9%), upper endoscopy with biopsy by 13 (15.1%), skin testing to evaluate sensitization by 4 (4.6%), and rectal biopsy by one respondent (1.2%). Although they were included in the alternatives, none of the respondents would request the following tests: contrast radiography of the esophagus, stomach and duodenum, occult blood in the stool, or measurement of α-1-antitrypsin in the stool. When the 140 respondents were asked about the first-choice formula they would prescribe for infants with suspected gastroesophageal reflux secondary to cow's milk allergy, the following answers were obtained: 70 (50.0%) would recommend formula with extensively hydrolyzed protein, 44 (31.4%) formula with soy protein isolate, 16 (11.4%) formula with partially hydrolyzed protein, 7 (5.0%) amino acid-based formula and 3 (2.1%) soy-based formula.

Table 3 shows the answers corresponding to clinical scenarios 1 and 2 according to the time of graduation from Medical School. There was a decrease in test requests as the years since graduation increased; however, no statistical significance was observed. For the other questions, the percentages were very similar, regardless of the year of graduation.

**Table 3:** Practice of pediatricians for a clinical scenario compatible with regurgitation in infants ("physiological reflux") and another clinical scenario compatible with gastroesophageal reflux disease in the first semester of life according to the time of graduation from medical school.

		Year of graduation in Medicine			
		Before 2000(n=51)	2000 to 2005(n=54)	After 2005(n=35)	p
Clinical scenario 1 (“physiological reflux”)					
Do you request any diagnostic test?	No	46 (90.2%)	49 (90.7%)	29 (82.9%)	0.469
Do you prescribe any medication?	No	42 (82.3%)	43 (79.6%)	29 (82.6%)	0.908
Do you recommend change in formula?	Yes	15 (29.4%)	15 (27.8%)	12 (34.3%)	0.801
Do you recommend a position to sleep?	Yes	46 (90.2%)	52 (96.2%)	34 (97.1%)	0.284
Clinical scenario 2 (GERD)					
Do you request any diagnostic test?	Yes	25 (49.0%)	32 (59.3%)	27 (77.1%)	0.097
Do you prescribe any medication?	Yes	43 (84.3%)	49 (90.7%)	30 (85.7%)	0.590
Do you recommend change in diet?	Yes	42 (82.4%)	38 (70.4%)	25 (71.4%)	0.313
Do you recommend a position to sleep?	Yes	48 (94.1%)	49 (90.7%)	33 (94.3%)	0.742

GERD, gastroesophageal reflux disease.

## Discussion

Based on the expected answers related to test requests and medication prescription, the results allow us to infer that most pediatricians adequately differentiate regurgitation in infants ("physiological reflux") from GERD (Table 1).

Regarding the required tests, there was a predominance of contrast x-ray of the esophagus, stomach and duodenum, which is traditionally regarded as the first test to be requested to rule out digestive tract anatomical abnormalities.^3,7^ Esophageal pH-metry also appears as a frequent request. In this case, it is likely to reflect knowledge more than practice itself, considering that in the past, this test was indicated in literature as the gold standard for the diagnosis of GERD. In this context, it should be remembered that, in daily clinical practice, esophageal pH monitoring is not always available and it is often not accepted by the family. Currently, for research purposes, esophageal pH-metry has been replaced by the impedance-pH monitoring test, which still has a number of problems to be solved before it can be routinely applied in clinical practice.^9^ In turn, ultrasonography was indicated in a few answers (Table 1). Indeed, ultrasonography is not considered useful in evaluating the infant with gastroesophageal reflux, although it allows the visualization of the stomach reflux into the esophagus, which can occur both in physiological reflux and GERD.^3^^,^ 5 On the other hand, ultrasonography is a good method for the diagnosis of hypertrophic pyloric stenosis.^3,5^ It should be noted that, according to the Rome III criteria,^2^ the diagnosis of regurgitation in infants should be established mainly based on clinical manifestations. It is characterized by the occurrence of two or more regurgitations a day for three or more weeks in the absence of nausea, hematemesis, aspiration, growth deficit and feeding and swallowing difficulties or abnormal posture.^2^

Another remarkable result is the intention to prescribe prokinetics, especially domperidone and bromopride, which would be present in the prescription of 18.6% of pediatricians when treating a case of regurgitation in infants and in 72.8% of prescriptions in a patient with a clinical picture compatible with GERD (Table 1). In the case of GERD, prokinetics would often be associated by respondents with medications for the reduction of gastric acid production. In the guideline of NASPGHAN/ESPGHAN,^3^ published in 2009, prokinetic agents are not recommended due to the lack of clinical evidence of their efficacy for the treatment of gastroesophageal reflux in infants. The guideline^3^ emphasizes there is no "robust" evidence for the indication of domperidone based on a systematic review published in England, which analyzed four studies published in the 1970s and 1980s.^10^ On the other hand, a meta-analysis carried out and published in Brazil, considering a broad definition for the outcome (change in reflux symptoms), concluded that domperidone has satisfactory clinical efficacy.^11^ It is noteworthy that both meta-analyses^10^^,^ 11 were practically based on the same clinical trials. It is not known why there are no more recent clinical trials with larger series, but still, in practice, domperidone continues to be prescribed, probably based on the individual experience of each professional. Recent evidence shows that domperidone may cause elongation of the ST segment on the electrocardiogram.^12^^-^ 15 A meta-analysis that evaluated the effect of metoclopramide in the treatment of reflux concluded there was no evidence of its efficacy or lack of efficacy.^16^ However, one should remember the extrapyramidal side effects that may be caused by metoclopramide and bromopride, and less frequently, by domperidone.^3^ Regarding the conducts for infants with GERD, it was observed that, in many cases, the interviewed pediatricians' answers disagreed with the recommendations of NASPGHAN/ESPGHAN of 2009.^3^ This disagreement may be due to the lack of knowledge of this new guideline on GERD or the difficulty not to medicate infants who have symptoms that effectively generate great concern for the family and the doctor himself.

A study carried out in 11 European countries, involving 567 pediatricians, showed that only 1.8% of them fully followed the recommendations of NASPGHAN/ESPGHAN of 2009.^17^ It also emphasized that 39% of the assessed European pediatricians prescribed proton-pump inhibitors for infants with unexplained crying and 36% for infants with regurgitation and vomiting not associated with complications. Thus, the use of proton-pump inhibitors with no precise indication is much higher among European pediatricians^17^ than among Brazilian ones, as shown in Table 1. 

It should be noted that most respondents would not recommend the supine position for an infant with physiological reflux or gastroesophageal reflux disease, which is recommended due to lower risk of sudden death.^3^ It is known that the supine position is not the one that provides greater reduction of reflux episodes, based on pH monitoring and impedance-pH monitoring; however, it is considered that the benefits of preventing sudden death are higher than those provided by the likely reduction in the manifestations of gastroesophageal reflux disease and gastroesophageal reflux.^3^^,^ 6 The American Academy of Pediatrics notes that the decrease in sudden infant death syndrome has been observed since 1992, when it was recommended that infants should not sleep in the prone position.^18^ It is noteworthy that 85% of 161 parents (aged 17 to 39 years) of infants, who were interviewed in a hospital in southwestern United States, according to an article published in 2012,^19^ believed that the supine position was the safest, and 60% of them chose to use it. Those who did not choose the supine position mentioned the child's preference or fear of choking or smothering.^20^ In Brazil, a case-control study^20^ compared a group consisting of 33 infants, victims of sudden death syndrome, with 192 living infants and 192 infants deceased from other causes. It can be verified that in this sample of the Brazilian population, the supine position was not adopted, as currently recommended worldwide,^18^ and in Brazil, by Pastoral da Criança and the Brazilian Society of Pediatrics.^20^

Regarding the prescription of changing the type of bottle for infants with probable clinical picture of "physiological reflux disease" or GERD, it was observed that, respectively, 30% and 75% of respondents recommended changes in the diet, generally recommending thickened formula or the addition of a thickener to the bottle. This conduct is in agreement with the NASPGHAN/ESPGHAN 2009 guideline.^3^ However, it is worth mentioning that in two clinical scenarios the possibility of diagnosis of reflux secondary to cow's milk allergy was not considered, although the alternatives include formulas with extensively hydrolyzed proteins and amino acid-based formula, as suggested in the NASPGHAN/ESPGHAN guideline.^3^ However, when the respondents were directly questioned about the possibility of gastroesophageal reflux disease being secondary to cow's milk allergy, most of them answered yes. When the 140 respondents were asked what formula was their first choice for an infant with suspected reflux secondary to cow's milk allergy at 5 months old, the following answers were obtained: 70 (50.0%) recommended formula with extensively hydrolyzed protein, 44 (31.4%) formula with soy protein, 16 (11.5%) formula with partially hydrolyzed protein, 7 (10.0%) amino acid-based formula and 3 (2.1%) soy-based formula. Currently, it is considered that infants with cow's milk allergy should receive formula with extensively hydrolyzed protein or amino acid-based formula as a substitute diet in the first semester of life.^3^^,^ 21 These data show an increase in the number of correct answers by pediatricians regarding substitutes for cow's milk for infants with cow's milk allergy when compared to that observed in a previous study,^22^ which showed that approximately 80% would prescribe soy formula and 40% soy extract, with the last option being inadequate to meet the nutritional requirements of infants. 

In conclusion, the results showed that the pediatricians have a good level of knowledge, compatible with the possibility of differentiating physiological reflux from GERD. The possibility of reflux secondary to allergy to cow's milk does not seem to be consistent with the guideline of NASPGHAN/ESPGHAN. It was also verified that many pediatricians would recommend positions different from the supine position (associated with increased risk of sudden death) and prescribe prokinetics, not in accordance with the guidelines published in 2009 by NASPGHAN/ESPGHAN.^3^
