# Supplementary material for: Knowledge and practice of Brazilian pediatricians on gastroesophageal reflux disease in infants
Source: Rev Paul Pediatr. 2015 Mar;33(1):12–8. doi: 10.1016/j.rpped.2014.11.005 (PMC4436951; doi:10.1016/j.rpped.2014.11.005)
Supplement: Translation

# Translation: Conhecimento e prática de pediatras brasileiros sobre a doença do refluxo gastroesofágico em lactentes

**Authors:** Ana Cristina Fontenele Soares, Carla Lima de Freitas, Mauro Batista de Morais

**Affiliations:** Universidade Federal de São Paulo (UNIFESP), São Paulo, SP, Brasil

**Keywords:** Refluxo gastroesofágico, Vômito, Lactente, Fórmulas infantis, Prática profissional, Conhecimentos, atitudes e prática em saúde

## Abstract

**OBJETIVO:**

Avaliar o conhecimento e a prática de pediatras brasileiros na assistência ao lactente com refluxo fisiológico e doença do refluxo gastroesofágico.

**MÉTODOS:**

Foram entrevistados 140 médicos pediatras em dois eventos científicos em 2009 e 2010. As perguntas referiam-se a dois casos clínicos de lactentes, um com quadro compatível com regurgitação do lactente (refluxo fisiológico) e outro com doença do refluxo gastroesofágico.

**RESULTADOS:**

Dos 140 participantes, 11,4% (n=16) e 62,1% (n=87) solicitariam exame para lactentes, respectivamente, com refluxo fisiológico e doença do refluxo gastroesofágico. O primeiro exame solicitado com maior frequência seria a radiografia contrastada de esôfago, estômago e duodeno. Medicação seria prescrita por 18,6% (n=26) para o caso de refluxo fisiológico e 87,1% (n=122) para o caso de doença do refluxo gastroesofágico. Procinéticos seriam prescritos com maior frequência do que os redutores da secreção ácida gástrica. Prescrição de posição para dormir fez parte das recomendações de 94,2% (n=132) e 92,9% (n=130) dos entrevistados, respectivamente, para os casos de refluxo fisiológico e doença do refluxo gastroesofágico. Entretanto, cerca da metade dos entrevistados não recomendaria o decúbito dorsal. Prescrição de dieta de exclusão do leite de vaca para um lactente com quadro de doença do refluxo gastroesofágico seria feita por apenas 10 (7,1%) dos participantes.

**CONCLUSÕES:**

Condutas diferentes das diretrizes internacionais são frequentemente consideradas adequadas, especialmente quanto à recomendação de posição diferente do decúbito dorsal e prescrição de medicamentos. As respostas permitem inferir a capacidade de correta diferenciação entre refluxo fisiológico e doença do refluxo gastroesofágico.

## Introdução

Vômitos e regurgitações ocorrem com frequência no lactente, em especial no primeiro semestre de vida.^1^ A maior parte dos casos se enquadra na regurgitação do lactente (refluxo fisiológico).^2^ ^and^ 3 Por outro lado, a doença do refluxo gastroesofágico (DRGE) caracteriza-se por manifestações clínicas variadas e inespecíficas não circunscritas obrigatoriamente ao aparelho digestório.^3^ O limite entre a regurgitação do lactente e a doença do refluxo gastroesofágico nem sempre é facilmente definido, sua diferenciação, com frequência, é um desafio na assistência ao lactente.^2^ ^and^ 3 Nesse contexto, existe uma preocupação crescente com a excessiva solicitação de exames complementares e de prescrições de medicamentos para lactentes saudáveis que apresentam regurgitações não ocasionadas pela doença do refluxo gastroesofágico.^2^^,^ 3 ^,^ 4 ^and^ 5 Por outro lado, a DRGE apresenta sintomatologia variada e, quando não conduzida de forma adequada, pode ocasionar morbidade.^2^^,^ 3 ^and^ 4

Diretrizes para a assistência do lactente com refluxo gastroesofágico vêm sendo publicadas nas últimas décadas com mudanças nas recomendações diagnósticas e terapêuticas.^2^^,^ 3 ^,^ 6 ^and^ 7 Em 2007, foi publicado um artigo com base em pesquisas feitas com profissionais da América do Norte que mostrou que, apesar da existência de várias diretrizes, muitos lactentes com refluxo gastroesofágico fisiológico continuam sendo tratados na América do Norte como se apresentassem DRGE.^8^

Considerando que pesquisas com profissionais permitem nortear programas de educação médica continuada, este estudo foi feito com o objetivo de avaliar o conhecimento e a prática de pediatras brasileiros na assistência ao lactente com refluxo fisiológico (regurgitação do lactente) e doença do refluxo gastroesofágico.

## Método

Os dados foram coletados em dois eventos científicos, em outubro de 2009 e em março de 2010. Foram entrevistados 140 médicos, 121 (86,4%) do gênero feminino, convidados ao acaso para participar da pesquisa. Com relação ao ano de graduação, 56 eram graduados após 2005, 34 entre 2000 e 2005 e 50 antes de 2000. De todos os participantes foi obtido consentimento por escrito, após esclarecimento sobre o objetivo da pesquisa. O projeto foi aprovado pelo Comitê de Ética em Pesquisa da Universidade Federal de São Paulo-Hospital São Paulo.

Com relação ao local de exercício profissional, 107 (76%) dos entrevistados atuavam na Região Sudeste, a maioria no Estado de São Paulo (66 na capital e 32 no interior). Os outros participantes eram do Nordeste (n=8; 6%), Sul (n=9; 6,5%), Norte (n=7; 5,5%) e Centro-Oeste (n=8; 6%).

As questões foram formuladas com base em cenários clínicos semelhantes aos usados previamente na literatura:^8^

- Cenário clínico 1 (expectativa de que fosse estabelecida a hipótese diagnóstica de regurgitação do lactente ou refluxo fisiológico'): "Vômitos involuntários de duas a quatro vezes ao dia em um lactente de dois meses em uso de fórmula infantil e que se apresentava no percentil 90 de peso e estatura. Sem outras manifestações clínicas". - Cenário clínico 2 (expectativa de que fosse estabelecida a hipótese diagnóstica de doença do refluxo gastroesofágico): "Lactente de cinco meses em uso de fórmula infantil. Apresenta frequentes regurgitações desde o nascimento e há dois meses apresenta quadro de irritabilidade e dificuldade de ganhar de peso. Apresentou melhoria parcial com medidas posturais".

Para cada um desses dois cenários clínicos foram formuladas as seguintes perguntas, solicitando que fossem respondidas de acordo com sua conduta prática: 1. Você solicitaria algum exame? Se a resposta for sim, qual o primeiro exame?; 2. Você prescreveria alguma medicação? Se sim, qual seria sua primeira prescrição; 3. Você modificaria a dieta? Se sim, qual sua primeira recomendação; 4. Você recomendaria qual posição no berço? Para todas as questões eram oferecidas opções conforme apresentado nas Tabela 1 and tabela 2 da seção de resultados. Na ficha de entrevista eram apresentados os nomes comerciais dos produtos dietéticos e medicamentos.

**Tabela 1:** Prática de pediatras para um cenário clínico compatível com regurgitação do lactente (refluxo fisiológico) e outro compatível com doença do refluxo gastroesofágico no primeiro semestre de vida quanto à indicação de exame e prescrição de medicamentos

	Cenário clínico 1: refluxo fisiológico (n=140)	Cenário clínico 2: DRGE (n=140)
**Você solicita algum exame? Qual o 1° exame?**		
*Não*	124 (88,6%)	53 (37,9%)
*Sim, qual o primeiro exame?*	16 (11,4%)	87 (62,1%)
RX contrastado do esôfago, estômago e duodeno	7 (43,8%)	48 (55,1%)
pHmetria esofágica de 24 horas	5 (31,3%)	30 (34,5%)
Estudo com radionucleotídeo (cintilografia)	1 (6,2%)	4 (4,6%)
Ultrassom para pesquisa de RGE	2 (12,5%)	2 (2,3%)
Endoscopia digestiva alta com biópsia	-	2 (2,3%)
Manometria esofágica	-	1 (1,2%)
RX simples de abdome	1(6,2%)	-

*Você prescreve alguma medicação?*		
*Não*	114 (81,4%)	18 (12,9%)
*Sim, qual a primeira prescrição?*	26 (18,6%)	122 (87,1%)
Metoclopramida	1 (3,8%)	-
Bromoprida	13 (50%)	28 (23%)
Domperidona	12 (46,2%)	43 (35,2%)
Bromoprida + ranitidina ou omeprazol	-	2 (1,6%)
Domperidona + ranitidina	-	24 (19,7%)
Domperidona + omeprazol	-	5 (4,1%)
Ranitidina	-	15 (12,3%)
Ompeprazol	-	4 (3,3%)
Lanzoprazol	-	1 (0,8%)

DRGE, doença do refluxo gastroesofágico.

**Tabela 2:** Prática de pediatras para um cenário clínico compatível com regurgitação do lactente (refluxo fisiológico) e outro compatível com doença do refluxo gastroesofágico no primeiro semestre de vida quanto à recomendação da posição no berço e mudança de fórmula

	Cenário 1: Refluxo fisiológico (n=140)	Cenário 2 DRGE (n=140)
**Você recomenda mudança na posição?**		
*Não*	8 (5,8%)	10 (7,1%)
*Sim, qual a posição?*	132(94,2%)	130 (92,9%)
Decúbito ventral	1 (0,7%)	3 (2,4%)
Decúbito ventral com elevação de 30°	29 (22,0%)	32 (24,6%)
Decúbito dorsal	4 (3,0%)	5 (0,3%)
Decúbito dorsal com elevação de 30°	61 (46,2%)	54 (41,5%)
Decúbito lateral direito	5 (3,8%)	3 (2,4%)
Decúbito lateral direito com elevação de 30°	7 (5,3%)	7 (5,4%)
Decúbito lateral esquerdo	8 (6,1%)	8 (6,1%)
Decúbito lateral esquerdo com elevação de 30°	17 (12,9%)	18 (13,8%)
Qualquer posição	-	-

**Você recomenda mudança de fórmula?**		
*Não*	98 (70%)	35 (25%)
*Sim, qual mudança?*	42 (30%)	105 (75%)
Adiciona espessante na mamadeira	8 (19%)	25 (23,8%)
Fórmula infantil antirregurgitação	33 (78,6%)	70 (66,7%)
Fórmula com proteína parcialmente hidrolisada	-	4 (0,3%)
Fórmula com proteínas extensamente hidrolisada	-	-
Fórmula com aminoácidos	-	-
Fórmula com proteína de soja	2 (0,4%)	6 (5,7%)

DRGE, doença do refluxo gastroesofágico.

Considerando a diretriz da Naspghan/Espghan^3^ (North American Society for Pediatric Gastroenterology, Hepatology and Nutrition/European Society for Pediatric Gastroenterology, Hepatology and Nutrition), publicada em 2009, que enfatiza a associação entre alergia ao leite de vaca e refluxo gastroesofágico em pediatria, foram formuladas as seguintes perguntas: "No lactente, a doença do refluxo gastroesofágico pode ser secundária à alergia proteína do leite de vaca?" Se a resposta for sim: 1. Solicita algum exame, se sim, qual o primeiro exame? 2. Lactente com menos de seis meses de idade, em uso de fórmula infantil, com suspeita de doença de refluxo gastresofágico secundária à alergia à proteína do leite de vaca, qual das seguintes opções dietéticas você prescreve inicialmente?

Na análise estatística foi usado o programa Epi-Info versão 3.2.2 (Atlanta, GA, EUA) para o cálculo do teste do qui-quadrado.

## Resultados

A tabela 1 apresenta os resultados obtidos para as perguntas relativas aos cenários clínicos 1 e 2 quanto à solicitação de exames e prescrição de medicamentos. Para um lactente com quadro compatível com refluxo fisiológico, 88,6% dos entrevistados não solicitariam exames e 18,6% prescreveriam uma medicação procinética. Para um lactente com quadro sugestivo de DRGE (cenário clínico 2), as proporções de entrevistados que solicitariam exames e prescreveriam medicamentos são maiores. O exame solicitado inicialmente, na maior parte das vezes, é a radiografia contrastada de esôfago, estômago e duodeno. Frente ao cenário compatível com DRGE, 87,1% dos entrevistados prescrevem medicamentos com predomínio de procinéticos e ranitidina.

Com relação à posição recomendada para manter a criança no berço (tabela 2), observa-se que 95% dos entrevistados preconizam mudança para ambos os cenários clínicos. No entanto, posições diferentes do preconizado na atualidade (decúbito dorsal) seriam recomendadas por número expressivo de pediatras (54,4% para o lactente com refluxo fisiológico e 42,1% para o lactente com quadro compatível com DRGE).

Fórmulas espessadas industrializadas ou o acréscimo de espessantes nas mamadeiras é a recomendação predominante. Destaca-se que a mudança no tipo de mamadeira é recomendada com maior frequência no cenário clínico correspondente à DRGE (tabela 2). Por outro lado, deve ser destacado que apenas dez entrevistados excluiriam a proteína do leite de vaca da dieta como primeira conduta dietética para o lactente com quadro compatível com doença do refluxo gastroesofágico (tabela 2).

Com relação à questão sobre a possibilidade da doença do refluxo gastroesofágico ser secundária à alergia ao leite de vaca, 105 (75%) dos entrevistados responderam que sim, 16 (11,4%) que não e 19 (13,6%) que são manifestações clínicas semelhantes, decorrentes, no entanto, de doenças diferentes. Dos 140 entrevistados, 86 (82%) solicitariam algum exame. São os seguintes o primeiro exame indicado: dosagem de IgE específica contra o leite de vaca por 44 (51,2%), dosagem sérica de IgE total por 24 (27,9%), endoscopia digestiva alta com biópsias por 13 (15,1%), teste cutâneo para avaliar sensibilização por quatro (4,6%) e biópsia retal por um único entrevistado (1,2%). Apesar de constar das opções das perguntas, nenhum dos entrevistados solicitaria os seguintes exames: radiografia contrastada de esôfago, estômago e duodeno, pesquisa de sangue oculto nas fezes ou dosagem de a-1-antitripsina nas fezes. Quando se perguntou aos 140 entrevistados qual a primeira fórmula que prescreveria para um lactente com suspeita de refluxo gastroesofágico secundário à alergia ao leite de vaca, obtiveram-se as seguintes respostas: 70 (50%) recomendariam fórmula com proteína extensamente hidrolisada, 44 (31,4%) fórmula com proteína isolada de soja, 16 (11,4%) fórmula com proteína parcialmente hidrolisada, sete (5%) fórmula de aminoácidos e três (2,1%) bebidas à base de extrato de soja.

A tabela 3 apresenta as respostas correspondentes aos cenários clínicos 1 e 2 de acordo com a época da graduação em medicina. Observou-se diminuição da indicação de exames quando maior o número de anos de graduação, no entanto, o estudo estatístico não atingiu significância. Para as demais perguntas os percentuais foram muito semelhantes independentemente do ano de graduação.

**Tabela 3:** Prática de pediatras para um cenário clínico compatível com regurgitação do lactente (refluxo fisiológico) e outro compatível com doença do refluxo gastroesofágico no primeiro semestre de vida de acordo com a época de graduação em medicina

		Ano de graduação em medicina			
		Antes de 2000	Entre 2000 e 2005	Após 2005	*p*
		(n=51)	(n=54)	(n=35)	
*Cenário clínico 1 (refluxo fisiológico)*					
Você solicita algum exame?	Não	46 (90,2%)	49 (90,7%)	29 (82,9%)	0,469
Você prescreve alguma medicação?	Não	42 (82,3%)	43 (79,6%)	29 (82,6%)	0,908
Você recomenda mudança da fórmula?	Sim	15 (29,4%)	15 (27,8%)	12 (34,3%)	0,801
Você recomenda posição para deitar?	Sim	46 (90,2%)	52 (96,2%)	34 (97,1%)	0,284

*Cenário clínico 2 (DRGE)*					
Você solicita algum exame?	Sim	25 (49%)	32 (59,3%)	27 (77,1%)	0,097
Você prescreve alguma medicação?	Sim	43 (84,3%)	49 (90,7%)	30 (85,7%)	0,590
Você recomenda mudança na dieta?	Sim	42 (82,4%)	38 (70,4%)	25 (71,4%)	0,313
Você recomenda posição para deitar?	Sim	48 (94,1%)	49 (90,7%)	33 (94,3%)	0,742

DRGE, doença do refluxo gastroesofágico.

## Discussão

Com base na expectativa de respostas relativas à solicitação de exames e prescrição de medicamentos, os resultados permitem inferir que a maioria dos pediatras distingue adequadamente a regurgitação do lactente (refluxo fisiológico) da DRGE (tabela 1). Com relação aos exames solicitados, observou-se predomínio da radiografia contrastada de esôfago, estômago e duodeno, que é considerada tradicionalmente como o primeiro exame a ser solicitado para descartar anormalidades anatômicas do tubo digestivo.^3^ ^and^ 7 A pHmetria esofágica aparece também como uma solicitação frequente. Nesse caso, é provável que reflita mais o conhecimento do que a prática propriamente dita, considerando que, no passado, esse exame foi indicado na literatura como padrão-ouro para o diagnóstico da DRGE. Nesse contexto, deve ser lembrado que, na prática clínica diária, a pHmetria esofágica nem sempre é disponível e com frequência não é aceita pelos pais. Atualmente, para fins de pesquisa, a pHmetria esofágica vem sendo substituída pelo impendâncio-pHmetria, que ainda tem uma série de problemas a ser em resolvidos antes da sua aplicação rotineira na prática clínica.^9^ Por sua vez, a ultrassonografia foi indicada em poucas respostas (tabela 1). Realmente, a ultrassonografia não é considerada útil na avaliação do lactente com refluxo gastroesofágico, apesar de permitir a visualização do refluxo do estômago para o esôfago que pode ocorrer tanto no refluxo fisiológico como na DRGE.^3^ ^and^ 5 Por outro lado, o ultrassom é um bom método para o diagnóstico de estenose hipertrófica do piloro.^3^ ^and^ 5 Deve ser ressaltado que, de acordo com o critério de Roma III,^2^ o diagnóstico de regurgitação do lactente deve ser estabelecido fundamentalmente com base nas manifestações clínicas. Caracteriza-se pela ocorrência de duas ou mais regurgitações diárias por três ou mais semanas na ausência de náuseas, hematêmese, aspiração, déficit de crescimento e dificuldades para alimentação e deglutição ou postura anormal.^2^

Outro resultado contundente é a intenção de prescrever procinéticos, especialmente a domperidona e a bromoprida, que estariam presentes na prescrição de 18,6% dos pediatras ao atender um caso de regurgitação do lactente e em 72,8% das prescrições de um paciente com quadro compatível com DRGE (tabela 1). No caso da DRGE, os procinéticos com frequência seriam associados pelos entrevistados com medicamentos destinados à redução da produção gástrica de ácido. Na diretriz da Naspghan/Espghan,^3^ publicada em 2009, os agentes procinéticos não são recomendados em função da falta de evidência clínica de sua eficácia para o tratamento do refluxo gastroesofágico do lactente. A diretriz^3^ destaca não existir evidência robusta para a indicação da domperidona com base em revisão sistemática publicada na Inglaterra que analisou quatro artigos publicados nas décadas de 1970 e 1980.^10^ Por outro lado, metanálise feita e publicada no Brasil, considerando uma definição ampla para o desfecho (modificação dos sintomas de refluxo), concluiu que a domperidona apresenta eficácia clínica satisfatória.^11^ Vale destacar que ambas as metanálises^10^ ^and^ 11 basearam-se praticamente nos mesmos ensaios clínicos. Não se sabe a razão pela qual não existem ensaios clínicos mais recentes e com casuísticas maiores, mesmo assim, na prática, a domperidona continua sendo prescrita, provavelmente com base na experiência individual de cada profissional. Evidências recentes mostram que a domperidona pode provocar alongamento do segmento QT no eletrocardiograma.^12^^,^ 13 ^,^ 14 ^and^ 15 Metanálise que avaliou o efeito da metoclopramida no tratamento do refluxo concluiu que não existem provas de sua eficácia ou de sua falta de eficácia.^16^ Entretanto, não se deve deixar de lembrar os efeitos colaterais extrapiramidais que podem ser provocados pela metoclopramida e bromoprida e, em menor frequência, pela domperidona.^3^ Com relação às condutas destinadas a lactentes com DRGE, observou-se que, em muitos casos, as respostas dos pediatras entrevistados discordam das recomendações da Naspghan/Espghan de 2009.^3^ Essa discordância pode ter ocorrido pela falta de conhecimento dessa nova diretriz sobre DRGE ou pela dificuldade de não medicar lactentes que apresentam sintomas que efetivamente geram grande preocupação para os pais e para o próprio médico. Artigo elaborado em 11 países europeus, que envolveu 567 pediatras, mostrou que apenas 1,8% deles segue plenamente as recomendações da Naspghan/Espghan de 2009.^17^ Destacam, ainda, que 39% dos pediatras europeus pesquisados prescrevem inibidores de bomba de prótons para lactentes com choro inexplicado e 36% para lactentes com regurgitação e vômitos não associados com complicações. Assim, o uso de inibidores de bomba de prótons sem indicação precisa é muito maior entre os pediatras europeus^17^ do que entre os brasileiros, conforme pode ser observado na tabela 1.

Deve ser destacado que grande parte dos entrevistados não recomendaria a posição em decúbito dorsal para um lactente com refluxo fisiológico ou doença do refluxo gastroesofágico, que é a recomendada por apresentar menor risco de morte súbita.^3^ Sabe-se que o decúbito dorsal não é a posição que proporciona maior redução dos episódios de refluxos com base na pHmetria e impedâncio-pHmetria, entretanto, considera-se que as vantagens por prevenir a morte súbita são maiores do que as proporcionadas pela provável redução nas manifestações da doença do refluxo gastroesofágico.^3^ ^and^ 6 A Academia Americana de Pediatria ressalta que se observou declínio na síndrome da morte súbita desde 1992, quando foi recomendado que os lactentes não dormissem na posição prona (decúbito ventral).^18^ É interessante que 85% de 161 pais (entre 17 e 39 anos) de lactentes entrevistados em um hospital do sudoeste dos Estados Unidos, de acordo com um artigo publicado em 2012,^19^ acreditavam que a posição supina fosse a mais segura e 60% deles a adotavam. Os que não usavam a posição supina mencionavam preferência da criança ou medo de engasgo ou sufocamento.^20^ No Brasil, estudo caso-controle^20^ comparou um grupo constituído de 33 lactentes vítimas da síndrome de morte súbita com 192 lactentes vivos e 192 lactentes falecidos de outras causas. Pode-se verificar que, nessa amostra da população brasileira, não se adotava o decúbito dorsal conforme o preconizado atualmente internacionalmente^18^ e, no Brasil, pela Pastoral da Criança e pela Sociedade Brasileira de Pediatria.^20^

No que se refere à prescrição de mudanças nas mamadeiras dos lactentes com quadro clínico provável de refluxo fisiológico ou de DRGE, constatou-se que, respectivamente, 30% e 75% dos entrevistados recomendariam mudança da dieta, em geral, com a preconização de fórmula espessada ou a adição de um espessante na mamadeira. Essa conduta é concordante com a diretriz da Naspghan/Espghan de 2009.^3^ No entanto, é interessante mencionar que, nos dois cenários clínicos, não foi considerada a possibilidade do diagnóstico de refluxo secundário à alergia ao leite de vaca, apesar de as opções incluírem as fórmulas com proteínas extensamente hidrolisadas e de aminoácidos como sugerido na diretriz da Naspghan/Espghan.^3^ No entanto, quando se perguntou diretamente sobre a possibilidade da doença do refluxo gastroesofágico ser secundária à alergia ao leite de vaca, grande parte dos entrevistados respondeu que sim. Quando se perguntou aos 140 entrevistados qual a primeira fórmula que prescreveria para um lactente com suspeita de refluxo secundário à alergia ao leite de vaca aos cinco meses, obtiveram-se as seguintes respostas: 70 (50%) fórmula com proteína extensamente hidrolisada, 44 (31,4%) fórmula com proteína isolada de soja, 16 (11,5%) fórmula com proteína parcialmente hidrolisada, sete (10%) fórmula de aminoácidos e três (2,1%) bebidas à base de extrato de soja. Atualmente, considera-se que os lactentes com alergia ao leite de vaca no primeiro semestre de vida devam receber como dieta substitutiva fórmula com proteína extensamente hidrolisada ou fórmula de aminoácidos.^3^ ^and^ 21 Esses dados mostram um incremento no índice de acertos dos pediatras a respeito dos substitutos do leite de vaca para lactentes com alergia ao leite de vaca em relação ao observado em um estudo anterior,^22^ que mostrou que cerca de 80% poderiam prescrever fórmula de soja e 40% extrato de soja, a última opção inadequada para suprir necessidades nutricionais de lactentes.

Em conclusão, os resultados mostraram que os pediatras entrevistados apresentam bom nível de conhecimento compatível com a possibilidade de diferenciar refluxo fisiológico de DRGE. A possibilidade de refluxo secundário a alergia ao leite de vaca não parece ser concordante com a diretriz da Naspghan/Espghan. Constatou-se, ainda, que muitos pediatras recomendariam posições diferentes do decúbito dorsal (associadas à maior risco de morte súbita) e prescrevem procinéticos, condutas não alinhadas com as diretrizes divulgadas em 2009 pela Naspghan/Espghan.^3^
